# Gut Microbiota Affects Mouse Social Behavior via Hippuric Acid Metabolism

**DOI:** 10.3390/neurolint17110185

**Published:** 2025-11-11

**Authors:** Momona Tsukui, Sosuke Yagishita, Shinji Tokunaga, Shuji Wakatsuki, Toshiyuki Araki

**Affiliations:** 1 Department of Peripheral Nervous System Research, National Institute of Neuroscience, National Center of Neurology and Psychiatry, 4-1-1 Ogawa-higashi, Kodaira 187-8502, Tokyo, Japan; 2 Department of Biotechnology and Life Science, Faculty of Engineering, Tokyo University of Agriculture and Technology, 2-24-16 Naka-Cho, Koganei-Shi 184-8588, Tokyo, Japan

**Keywords:** autism spectrum disorder (ASD), germ-free, hippuric acid, oxytocin, hypothalamus

## Abstract

Background/Objectives: Autism spectrum disorder (ASD) is a neurodevelopmental disorder typically characterized by impaired social communication. Previous reports have postulated gut microbiota to be an important non-genetic factor affecting ASD-like phenotypes in mice, as germ-free (GF) mice show impaired social communication. Results: In this study, we identified hippuric acid (HA) as a metabolite generated via a gut microbiome-dependent mechanism that plays a role in the acquisition of social behavior during mouse development. We discovered that oral or intraperitoneal HA administration to GF mice normalizes their social behavior. Furthermore, HA administration restored oxytocin expression in the hypothalamic paraventricular nucleus and secretin expression in the subfornical organ, suggesting that HA may activate the secretin–oxytocin system to influence the social behavior of mice. Conclusions: These findings indicate that HA may serve as an important gut microbiome-dependent mediator affecting the brain mechanisms involved in regulating social behavior.

## 1. Introduction

Impaired social behavior is a typical feature that characterizes autism spectrum disorder (ASD), a neurodevelopmental disability with increasing recognition in society [1,2]. Genetic and environmental factors are involved in the development of social behavior [3,4], but the detailed mechanisms are still unknown. Mice bearing mutations or deletions in the genes responsible for diseases manifesting ASD-like social behavior abnormalities in humans share the phenotype of disinterest in other individuals, which is identified as ASD-like social behavior disorder in mice [5]. These genes include the neuroligin family of synaptic cell adhesion proteins and the Shank family of postsynaptic scaffolding proteins, both of which play essential roles in synaptic development and function [5]. Among them, Shank3 is a well-studied component of the postsynaptic density (PSD) in excitatory synapses and interacts with various synaptic molecules [6]. Multiple human genetic studies have established a connection between *SHANK* mutations and ASD, and *shank3* mutant mice exhibit impaired social interactions.

Because gut microbiome is altered in the mice exhibiting the ASD-like social behavior deficits [7] and germ-free (GF) mice exhibit ASD-like social behavioral deficits [8], the gut microbiome is thought to be an environmental factor influencing social behavior development. Among the possible mechanisms by which the gut microbiome influences social behavior, metabolites derived from gut bacteria have been identified to play a key role [9,10]. Gut bacteria can produce metabolites via their own metabolic pathways or by influencing host metabolism. Metabolites derived from gut bacteria are thought to influence host behavior by crossing the intestinal mucosal barrier, entering the brain through the blood–brain barrier, or inducing the production of inflammatory cytokines through their effects on immune cells [11].

Oxytocin plays an important role in the regulation of social behavior [12,13]. In several genetically modified mouse models of ASD (i.e., mice in which a gene—whose human ortholog is recognized as an ASD-causal gene—has been mutated, resulting in abnormalities in social behavior), the number of oxytocin-positive cells has been found to be decreased in specific brain regions [14,15,16]. Furthermore, it has been shown that the number of oxytocin-positive cells in the hypothalamic paraventricular nucleus (PVN) is decreased in GF mice [7], and administration of *Lactobacillus reuteri* restores the number of oxytocin-positive cells in several ASD mouse models to a level similar to that in wild type, thereby restoring normal social behavior [14,15,16]. These findings suggest gut microbiota affects social behavior in mice via regulation of oxytocin levels in the PVN; however, the mechanism by which gut microbiota increases oxytocin levels in the PVN and other brain regions remains unclear. Secretin, a neuropeptide widely distributed in the brain, has also been found to be associated with social behavior [17,18]. Furthermore, secretin administration increases oxytocin expression in the PVN [19,20], and oxytocin treatment restores normal social behavior in secretin receptor KO mice [19], suggesting that the secretin–oxytocin system may be associated with social behavior.

In this study, with the aim of identifying how gut microbiota affects social behavior, we focused on metabolites derived from gut microbiota known to be important in the acquisition of social behavior using antibiotic-treated and GF mice.

## 2. Materials and Methods

### 2.1. Animals

All the experimental procedures using mice were approved by the Committee on Ethical Issues in Animal Experiments at the National Center of Neurology and Psychiatry (approval number: 2023007). C57BL/6J specific pathogen-free (SPF) and GF mice were purchased from Clea Japan, Tokyo, Japan. Wild-type mice (C57BL/6J), wild-type GF mice (C57BL/6), and Shank3c KO mice were housed at 25 ± 1 °C in a 12 h light/dark cycle. GF mice were maintained in an isolator chamber (No.10 CL-1003, Clea Japan). Male mice, after reaching 10 weeks of age, were used for behavioral and histological analysis. Shank3 KO mice were obtained from Dr. Shigeo Uchino (Teikyo University, Japan). In the antibiotics administration experiments and HA administration experiments, wild-type male SPF mice or GF mice with no drug administration served as controls. The number of mice in each set of experiments is described in the figure legends.

### 2.2. Social Novelty and Social Preference Test

The testing apparatus consisted of a rectangular, three-chambered box with an infrared video camera fitted on the lid (O’Hara & Co., Tokyo, Japan). Each chamber measured 20 × 40 × 22 cm, and the dividing walls were made from clear Plexiglas, with small rectangular openings (5 × 3 cm) allowing access into each chamber. Each test consisted of three consecutive sessions: habituation, sociality test, and social novelty test. In the habituation session, the subject mouse was placed in the middle chamber and allowed to explore the entire test box for 10 min. For the social novelty test, an unfamiliar male mouse (stranger 1) was placed in one of the side chambers, and the subject mouse was allowed to explore for 5 min. For the social preference test, a second unfamiliar male mouse (stranger 2) was placed in the empty chamber, and the subject mouse was allowed to explore for another 5 min. Data acquisition and analysis were performed automatically with the use of TimeCSI software (ver1.6.2.4; O’Hara & Co., Tokyo, Japan).

### 2.3. Administration of Antibiotics and Hippuric Acid (HA)

For oral administration of antibiotics, ampicillin sodium (1.0 g/L; Fujifilm Wako Pure Chemical Co., Osaka, Japan), neomycin sulfate (1.0 g/L; Fujifilm Wako Pure Chemical Co.), or vancomycin HCl (0.5 g/L; Fujifilm Wako Pure Chemical Co.) was dissolved in distilled water and used as drinking water of SPF mice at 7 weeks of age for 3~5 weeks, following a previous report [21]. Ampicillin and neomycin are typically active against Gram-negative bacteria, while vancomycin is active against Gram-positive bacteria [22]. The antibiotic administration did not significantly affect body weight or motility of the mice compared with untreated control mice. For oral administration of HA, sodium HA (Fujifilm Wako Pure Chemical Co.) was dissolved in distilled water, either at 0.36 g/L (which corresponds to approximately 100 mg/kg body weight/day of HA (high dose)) or 0.036 g/L (approximately 10 mg/kg body weight/day of HA (low dose)), and used as drinking water for subject mice at 7 weeks of age for 3 weeks. Mice in the experimental groups are supplied only with drinking water containing the experimental reagent. We confirmed that this HA concentration does not affect general mouse activity. Distilled water without HA or antibiotics was used for the untreated control.

For intraperitoneal administration of HA, the HA was dissolved in PBS at 8.4 g/L, and 300 μL of the HA solution (100 mg/kg body weight) was injected intraperitoneally daily to subject mice at 9 weeks of age for 1 week. PBS without HA was injected for the untreated control.

### 2.4. Metabolome Analysis

Fecal samples were collected from Control, Amp, Neo, Vanco-treated, and GF mice at 10 weeks of age (*n* = 1 for each condition). The collected fecal samples (30~50 mg) were dissolved in 500 μL distilled water, and centrifuged at 1000× *g* for 5 min to collect the supernatant. Protein was removed from the supernatant by centrifugation (9100× *g*, 4 °C, 1 h) using an ultrafiltration device (Human Metabolome Technologies (HMT), Yamagata, Japan), and preserved in −80 °C freezer. The frozen samples were sent to HMT for differential metabolome analysis. HMT used the CE-FTMS, an analyzer that combines capillary electrophoresis (CE) and Orbitrap, a Fourier Transform Mass Spectrometer (FTMS), to analyze cationic and anionic metabolites. From the detected peaks, peaks with a signal/noise (S/N) ratio of 3 or higher were automatically extracted, and the mass-to-charge ratio (*m*/*z*), peak area value, and migration time (MT) were obtained to determine all compounds. Principal component analysis of the obtained data was performed using the weighted UniFrac distance matrix.

### 2.5. HA Quantification by HPLC

Quantification of HA from blood and fecal samples was performed according to a previous report with minor modifications [23,24]. Blood serum samples were collected from the hearts of euthanized mice using 100% carbon dioxide gas and deproteinized with acetonitrile and chloroform. Fecal samples were dispersed in 0.1× D-PBS (50 μL per mg feces), homogenized using BioMasher II with PowerMasher II (NIPPI, Tokyo, Japan), and deproteinized. Analysis was performed on HPLC using a 4.6 250 mm C8 column (CERI, 722081, Tokyo, Japan) and UV detection at 254 nm (SHIMADZU, Kyoto, Japan; SPD-20A). Buffer flow was 1 mL/min using acetonitrile and ammonium acetate (20 mM, pH 5.0) with a gradient from 0% acetonitrile/100% ammonium acetate to 80% acetonitrile/20% ammonium acetate during 25 min. A calibration curve was prepared by running HA standards (30 nM~100 μM) and measuring the peak area.

### 2.6. Immunohistochemistry

Mice were euthanized by deep anesthesia using pentobarbital (100 mg/kg body weight) and transcardially perfused with 250 mL of 4% paraformaldehyde/PBS, and the brains were post-fixed overnight in the same fixative, followed by immersion in 30% sucrose/PBS for 3 days. Coronal brain sections were cut at 12–20 μm thick on a cryostat (HM550, Thermo Fisher Scientific, Waltham, MA, USA), and every other section was collected to be mounted on glass slides (CREST-coated, Matsunami, Kishiwada, Japan). Sections were washed 3 times with PBS and immersed in 5% normal goat serum/0.3% Triton X-100 in PBS for 1 h. The sections were then incubated with primary antibody overnight at 4 °C, followed by secondary antibody for 2 h at room temperature. The primary antibodies and their dilutions used in this work were as follows: rabbit anti-oxytocin (1:2000, ImmunoStar, Hudson, WI, USA; #20068) and rabbit anti-secretin (1:2000, Atlas Antibodies, Stockholm, Sweden; #HPA050961). For detection, goat anti-rabbit Alexa-Fluor 488 (1:300, Invitrogen, Waltham, MA, USA/Thermo Fisher Scientific, #A11008) was used, or HRP-conjugated anti-rabbit IgG (1:300, ROCKLAND immunochemicals, Limerick, PA, USA, #18-8816-31) followed by a TSA Plus Cyanine 3 System (AKOYA BIOSCIENCES, Marlborough, MA, USA, NEL74001KT) for amplification of the immunohistochemical signal. Fluorescent signals were captured on a Leica DMI6000 B microscope (Wetzlar, Germany), and analyzed using ImageJ (version: 1.53k) or ImageJ Fiji (version: 1.54f).

### 2.7. Statistical Analysis

The results were expressed as the mean ± SEM. Statistical analyses were performed using GraphPad Prism 8 software. Significant differences between groups were examined using Student *t*-test, one-way analysis of variance (ANOVA), Kruskal–Wallis ANOVA, or two-way repeated measures (RM) ANOVA (Tukey’s test, Dunn’s test, or Sidak’s test was used for post hoc comparison).

## 3. Results

### 3.1. Oral Administration of Ampicillin or Neomycin, but Not Vancomycin, Affects Social Behavior in Mice

**Figure 1:**
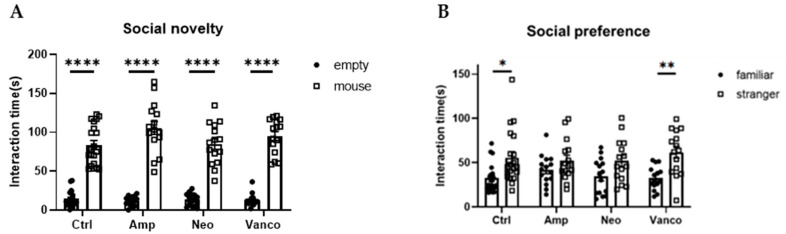
Social preference of mice is maintained after oral administration of vancomycin. Social novelty (**A**) and social preference (**B**) phases of the three-chambered social approach test. Time spent in proximity of empty and mouse cages (**A**), or familiar and stranger mouse cages (**B**), by the mice orally treated with indicated antibiotics is plotted on the y-axis (*n* = 20 for ctrl; *n* = 15 for Amp, Neo, and Vanco). Data are presented as mean ± SEM. *p*-values from two-way RM ANOVA followed by Sidak’s post hoc test. * *p* < 0.05, ** *p* < 0.01, **** *p* < 0.001.

Previous reports have shown that GF mice and specific pathogen-free (SPF) mice orally administered with a cocktail of antibiotics covering most microorganisms show impaired social behavior [7,8]. In our testing environment, lower preference for social subjects (stranger mice) than for non-social subjects (familiar mice) in the social novelty phase (social novelty test) was observed in GF mice and SPF mice orally treated with a cocktail of antibiotics (ampicillin, neomycin, and vancomycin), compared with untreated SPF mice, while social preference behavior (preference for stranger mice than empty cage) was similarly observed in mice of all conditions (Supplementary Figure S1). To gain insights into the effect of individual antibiotics on social preference, we orally administered ampicillin (Amp), neomycin (Neo), or vancomycin (Vanco) to SPF mice and measured the time spent searching for new individuals rather than known individuals (social novelty test). We found that the social preference is observed in the mice treated with Vanco, but not in mice treated with either Amp or Neo (Figure 1). These results suggest that social preference may be related to the maintenance of the gut bacteria susceptible to specific antibiotics.

### 3.2. Hippuric Acid Is Produced via Gut Flora-Mediated Metabolism

To identify factors involved in the social preference behavior alteration, we performed a comprehensive metabolomic analysis of fecal samples obtained from control, antibiotic-treated, and GF mice. To correlate the social preference trait with changes in gut metabolites, we screened the compounds detected more abundantly in mice showing the social novelty preference trait (i.e., untreated control and Vanco-treated mice) than in mice that lack social novelty preference (i.e., Amp-treated, Neo-treated, and GF mice). For this purpose, we obtained the level of each metabolite in fecal samples from the Amp-treated mice (shown as [Amp] for each compound in Table 1), Vanco-treated mice (shown as [Vanco]), Neo-treated mice (shown as [Neo]), and GF mice (shown as [GF]) relative to that from control mice (shown as [Control]). Values below the detection limit were calculated as 0. Subsequently, we calculated the value of ([Control] + [Vanco]) − ([Amp] + [Neo] + [GF]) for each compound to search for metabolites whose levels are correlated with social novelty preference behavior (Table 1). Among the list of metabolites, we chose to focus on hippuric acid (HA), which is detected only in the group maintaining social preference, and has previously been reported as a dysregulated metabolite in ASD patients, as well as in ASD model mice [25,26].

### 3.3. HA Administration Restores Social Novelty Behavior in GF Mice

**Figure 2:**
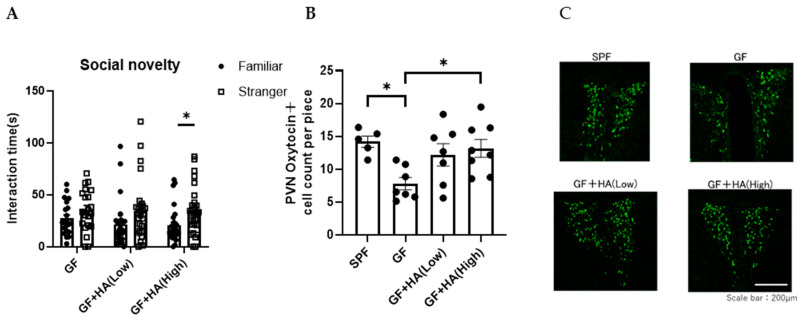
Oral HA administration restores the social preference of GF mice. GF mice were orally treated with HA. (**A**) Three-chambered social preference test. Time spent in proximity of familiar and stranger mouse cages by GF mice treated with HA is plotted on the y-axis (*n* = 21–28 per group). Data are presented as mean ± SEM. *p*-values are from two-way RM ANOVA followed by Sidak’s post hoc test. * *p* < 0.05. (**B**) The number of oxytocin-positive neurons in the PVN of the hypothalamus in GF mice orally treated with HA (*n* = 5–8 per group). Data are presented as mean ± SEM. *p*-value from one-way ANOVA with Tukey’s multiple comparison test. * *p* < 0.05. (**C**) Representative photomicrographs of oxytocin-positive neurons in the PVN of the hypothalamus in GF mice orally treated with HA. Scale bar = 200 μm.

**Figure 3:**
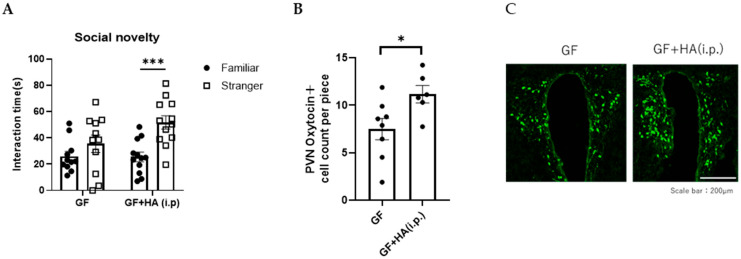
HA may be absorbed from the gut to the blood, affecting social novelty preference behavior. (**A**) Three-chambered social preference test. Time spent in proximity of familiar and stranger mouse cages by GF mice intraperitoneally treated with HA is plotted on the y-axis. (**B**) The number of oxytocin-positive neurons in the PVN of the hypothalamus in GF mice intraperitoneally treated with HA. Data are presented as mean ± SEM (*n* = 6–8), and the *p*-value from the control levels was determined by Student’s *t*-test. (* *p* < 0.05, *** *p* < 0.001). (**C**) Representative photomicrographs of oxytocin-positive neurons in the PVN of the hypothalamus in GF mice intraperitoneally treated with HA. Scale bar = 200 μm.

**Figure 4:**
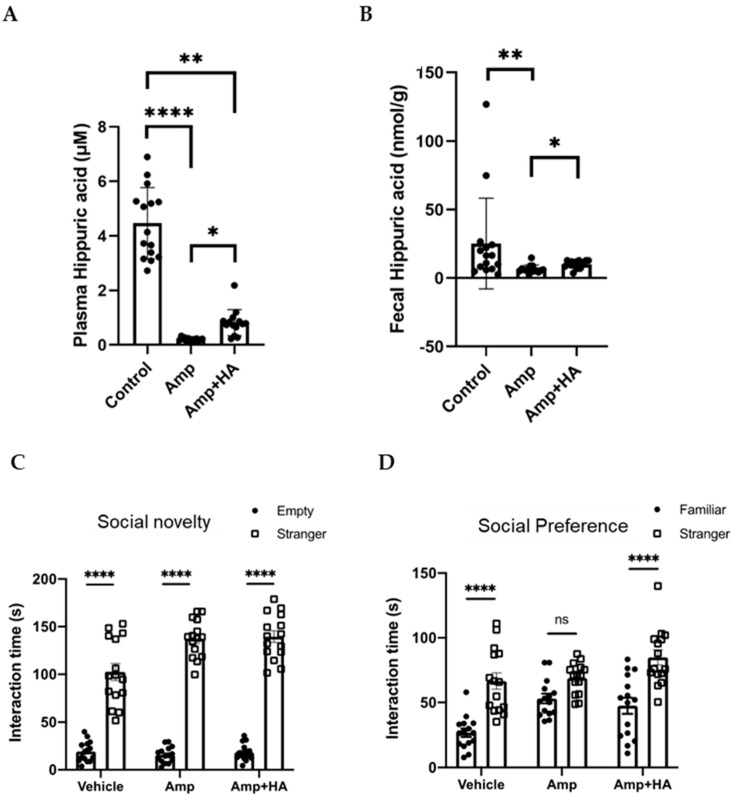
Oral HA administration restores social preference behavior of ampicillin-treated mice by increasing HA levels in feces and plasma. Ampicillin-treated mice were orally administered with HA. Serum (**A**) and fecal (**B**) HA concentrations of control and ampicillin-treated mice with/without oral HA administration. Untreated wild-type SPF mice served as controls. Sociality novelty (**C**) and social preference (**D**) phases of the three-chambered social approach test. Time spent in proximity of empty and mouse cages (**C**), or of familiar and stranger mouse cages (**D**) by ampicillin-treated mice orally administered with HA is plotted on the y-axis. *n* = 15 for ctrl, *n* = 14 for Amp, and *n* = 14 for Amp + HA. Data are presented as mean ± SEM. * *p* < 0.05, ** *p* < 0.01, **** *p* < 0.001.

To directly evaluate the role of HA on social preference, we orally administered HA to GF mice and examined the effect on their social preference. We found that the social preference is restored in GF mice treated with 0.36 g/L HA, suggesting that orally administered HA could replicate the effects of gut flora-derived factors on social preference (Figure 2A). To gain insights into how HA affects behavior, we administered HA to GF mice by intraperitoneal injection and examined their social preference. We found that intraperitoneal injection of HA is equally effective in restoring social novelty preference of GF mice (Figure 3A). These results suggest that orally administered HA is absorbed into the blood and exerts its effect on social behavior. To further confirm this hypothesis, we orally administered HA to ampicillin-treated mice and assessed social preference behavior and HA levels in feces and serum. We found that HA administration via drinking water (at 0.36 g/L) significantly elevated HA to detectable levels in both feces and serum (Figure 4A,B). In correlation with these increased HA levels, we found restored social preference behavior in HA-administered mice (Figure 4C,D). These results collectively suggest that HA generated by a gut flora-dependent mechanism affects social novelty preference behavior of mice by absorption from the gut to serum.

### 3.4. HA Administration Increases Secretin Production to Normalize Oxytocin Production in GF Mice

Oxytocin is a neuropeptide that plays a crucial role in social behavior and is implicated in ASD [12,13]. Previous reports have demonstrated that oxytocin treatment can reverse social deficits in several ASD mouse models [27]. Oxytocin is mainly synthesized in the hypothalamic paraventricular nucleus (PVN) of the brain [12,13], projecting its axons to the posterior pituitary, where the peptides are stored in vesicles until action potentials trigger their release. Thus, to investigate how HA influences social behavior in mice, we first focused on the effect of HA on oxytocin expression in the PVN. We found that the number of oxytocin-expressing neurons in the PVN is significantly decreased in GF mice compared to SPF mice, and the number was normalized by oral or intraperitoneal HA administration (Figure 2B,C and Figure 3B,C). These results suggest that HA may be involved in the production of oxytocin in the hypothalamus.

**Figure 5:**
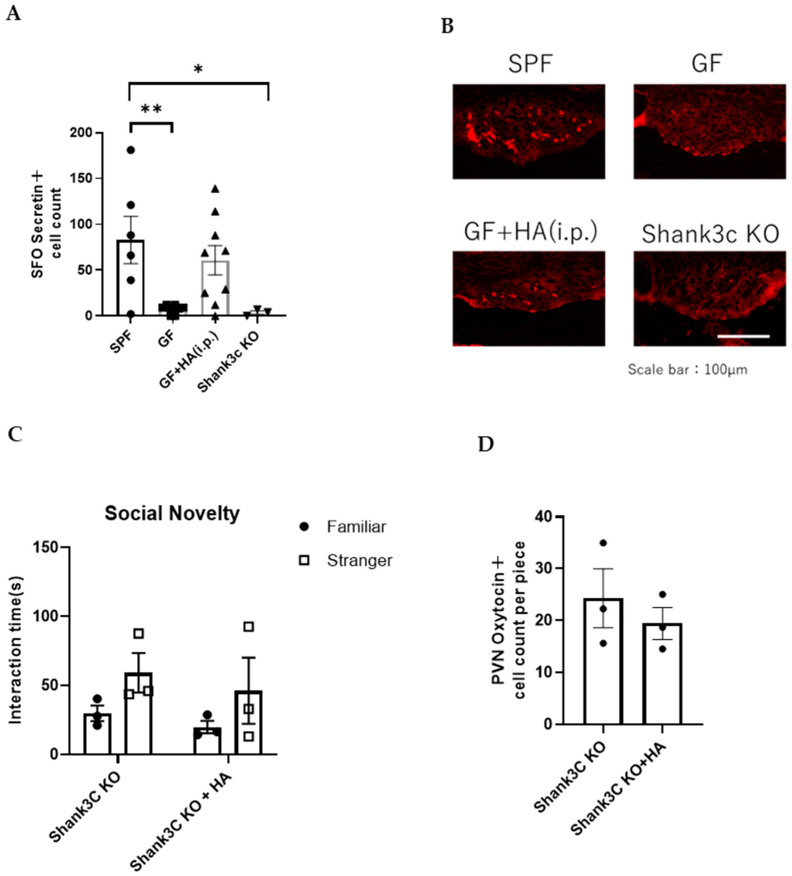
HA administration increases secretin expression in the PVN of GF mice. (**A**) The number of secretin-positive cells in SFO in the indicated mouse types. (*n* = 3–9 per group) Data was analyzed using Kruskal–Wallis ANOVA with post hoc Dunn’s test. * *p* < 0.05 and ** *p* < 0.01 indicates a statistical difference compared to the SPF group. (**B**) Representative photomicrographs of secretin-positive cells in SFO in indicated mouse types. Scale bar = 100 μm (**C**) Three-chambered social approach test. Time spent in proximity of familiar and stranger mouse cages by *Shank3* KO mice with or without intraperitoneal HA administration is plotted on the y-axis (*n* = 3 per group). (**D**) The number of oxytocin-positive neurons in the PVN of *Shank3* KO mice with or without intraperitoneal HA administration (*n* = 3 per group).

Secretin, a 27–amino acid peptide that is synthesized in various regions of the brain and duodenal S-cells in the gut, has also been implicated in the control of social behaviors [28]. Previous reports have shown that oxytocin expression in the PVN is induced by secretin administration [29,30] and that PVN receives secretin-mediated projections from the subfornical organ (SFO) [31]. Roles of secretin in regulating social behavior have also been demonstrated in previous works [32]. For instance, it was demonstrated by using a secretin receptor-deficient mouse model that secretin modulates social behavior [18]. To analyze how HA affects oxytocin production in the hypothalamus, we examined secretin expression in the SFO. We found that secretin expression in the SFO is decreased in GF mice, but normalized by HA treatment (Figure 5A,B). These findings suggest that HA administration may elevate secretin levels in the SFO and thereby lead to increased oxytocin expression in the PVN.

To investigate whether decreased secretin in the SFO is associated with impaired social behavior, we examined the number of secretin-positive cells in the SFO of other ASD model mice, in which molecules for synaptic signaling and/or development/maintenance are genetically modified. Among them, we chose to employ null mutant mice for *Shank3c*, a well-studied PSD component in excitatory synapses [33]. We found that the number of secretin-positive cells in SFO was significantly decreased in *Shank3c* KO mice compared to wild-type SPF mice (Figure 5A,B). This suggests that changes in secretin levels in the SFO may be associated with the social behavior of *Shank3c* KO mice. To examine whether HA administration can normalize social behavior abnormalities of Shank3c KO mice, we administered HA intraperitoneally to *Shank3c* KO mice using the same time schedule as the one used for HA administration to GF mice described above. We found that the impaired social behavior of *Shank3c* KO mice was not improved by HA administration (Figure 5C; *p* = 0.3318 for Familiar vs. Stranger comparison using *Shank3c* KO mice, and *p* = 0.4501 using HA-treated *Shank3c* KO mice by Two-way ANOVA). We also found that the number of oxytocin-positive cells in the hypothalamus was not significantly changed by HA administration (Figure 5D; *p* = 0.4925 by Student’s *t*-test). These results suggest that the mechanism of oxytocin expression reduction in *Shank3c* KO mice may be different from that in GF mice, or that *Shank3c* expression-mediated signaling may be necessary for oxytocin induction by HA administration.

## 4. Discussion

Oral administration of antibiotics to alter or eliminate gut microbiota typically involves a cocktail of multiple antibiotics with different antibacterial spectra. This approach effectively depletes the gut microbiota, mimicking the conditions observed in germ-free (GF) mice. Here, we administered individual antibiotics to analyze factors affecting social behavior lost in GF mice. Using this approach, we discovered that mice treated with vancomycin exhibit normal social novelty preference comparable to SPF mice. This finding led us to identify HA as a promising candidate for treatment.

HA is a well-known indicator in the field of occupational health because it is detected in the urine of individuals exposed to toluene [34,35]. It is known that dietary intake of benzoic acid, which is present in certain acidic foods (e.g., berries, plums, cranberries, and prunes), may influence urinary HA concentration and interfere with test data [36]. These data, together with our current results, suggest that the gut microbiota has a metabolic mechanism to actively generate HA. While HA is commonly recognized as a metabolite marker, it may also have a biological role. However, little is known about its potential bioactivity, particularly in the nervous system. The present findings provide the first evidence for the biological activity of HA in the brain mechanisms underlying the regulation of social behavior.

Previous studies have reported that microbiota-derived molecules and metabolites may affect host feeding and social behavior by modulating the activity of proopiomelanocortin and/or neuropeptide Y neurons in the hypothalamic arcuate nucleus [37]. This nucleus is located outside the blood–brain barrier (BBB) and is accessible to systemically circulating signaling molecules [38]. These arcuate neurons are known to regulate oxytocin production in the paraventricular nucleus (PVN) [39]. Interestingly, we found that HA administration can rescue secretin expression in the subfornical organ (SFO), which, like the arcuate nucleus, lacks a complete BBB [40]. Therefore, the mechanism whereby secretin regulates social behavior through the known pathway of oxytocin induction represents a biochemically and anatomically plausible cascade. Our findings suggest that secretin neurons in the SFO may also be influenced by gut bacteria-derived metabolites such as HA, ultimately affecting hypothalamic oxytocin production.

Previous reports have studied alteration of gut flora composition in different genetically modified ASD mouse models, including *Shank3c* KO. Intervention to gut flora by transferring specific bacteria or administering metabolites derived from gut flora shows therapeutic effects on social behavior abnormalities [41]. For instance, administration of 5-aminovaleric acid, which is found significantly lower in ASD mouse models, can normalize the social behavior of the mice by reducing neuronal excitability [8]. On the other hand, here we show that HA can normalize the social novelty preference behavior of GF mice, but not *Shank3c* KO mice. From these data, it is likely that the mechanisms underlying the reduction in secretin differ fundamentally between the two models. In GF mice, a decrease in gut bacteria-derived HA may lead to reduced secretin expression, resulting in diminished oxytocin signaling and impaired social behavior. In contrast, in Shank3c KO mice, intrinsic abnormalities at the synaptic or transcriptional level due to gene deletion may cause a reduction in secretin-positive cells independently of HA, which could explain the absence of behavioral or secretin recovery following HA administration. The HA–secretin–oxytocin pathway represents only one of multiple possible gut–brain communication routes influencing social behavior, and it is not proposed as a universal mechanism explaining ASD as a whole.

We have not been able to identify the specific bacteria responsible for HA production in the gut, thus far. In previous reports, HA production in the gut has been associated with *Clostridium sporogenes* [22,23] or *Eggerthella lenta* [41]. However, these bacteria are not likely to be responsible for HA production because they are Gram-positive bacteria, for which vancomycin exhibits antibacterial properties. Other reports have shown that HA can be generated from quinic acid in the gut [42]. Quinic acid may be metabolized to either shikimic acid or HA, depending on the gut microbiota status. We found, in our metabolomic analysis, that the levels of HA are higher in the mice showing social preference, while those mice with higher shikimic acid are in the group showing no social preference. These results suggest that the changes in HA observed in this study may be due to the bacteria, which are responsible for the metabolism of quinic acid to HA. Further studies are needed to determine the exact bacteria involved in this metabolic pathway and responsible for HA production.

### Limitations of This Study

While deficits in social behavior constitute a characteristic feature of ASD-like behavior, we recognize that, compared with other environmental models such as the maternal immune activation model or the valproic acid exposure model, GF mice are not considered a standard model of ASD and do not recapitulate the full spectrum of ASD pathology. In the manuscript, we therefore refer to them not as an ASD model but as a gut microbiota-dependent model of social behavioral abnormalities. To explore the possibility that the specific behavioral and molecular changes observed in GF mice reflect certain aspects of the social dysfunctions common to ASD, we focused on elucidating the mechanisms by which gut-derived metabolites regulate social behavior through neuroendocrine pathways.

While the current data postulate HA as a potential modifier for the development of social behavior, this does not necessarily mean that these findings can be directly transferred to human ASD. In particular, the finding that HA does not affect the social behavior of Shank3 KO mice suggests that HA may affect a specific subgroup of ASD in which gut microbiota abnormalities are involved.

## Figures and Tables

**Table 1:** List of compounds differentially present in the mouse gut administered with antibiotics.

Compound Name	Ctrl	Vanco	Amp	Neo	GF	Value
hippuric acid	1	8.2	0	0	0	4.6
syringic acid	1	3.8	0.32	0	0.44	2.15
N-formylmethionine	1	3.9	0	0.57	0.54	2.06
β-Ala-Lys	1	3.9	0	0.57	0.54	1.72
γ-butylbetaine	1	2.1	0.072	0.73	0.032	1.25
1-methylhydantoin glycine anhydride	1	1.4	0.35	0	0	1.1
N-acetylphenylalanine	1	2.1	0.34	0.5	0.58	1.08
p-aminophenol m-aminophenol	1	0.94	0	0	0	0.47
3-methylguanine	1	1.2	0.4	0.38	0.1	0.79
pyroglutamic acid	1	1	0.26	0.65	0	
trimethylamine	1	0.59	0.26	0	0.13	0.67
homoserine	1	0.63	0.15	0.36	0	0.64
alanine	1	0.84	0.16	0.45	0.49	0.55
ornithine	1	0.29	0.11	0.14	0.042	0.55
urocanic acid	1	0.26	0.083	0.13	0.14	0.51
aspartate	1	1.1	0.41	0.9	0.49	0.291667

## Data Availability

The original contributions presented in this study are included in the article/Supplementary Material. Further inquiries can be directed to the corresponding author.

## Supplementary Materials

The following supporting information can be downloaded at https://www.mdpi.com/article/10.3390/neurolint17110185/s1, Figure S1: Social behavior of germ-free mice.
