# Supplementary material for: Gut Microbiota Affects Mouse Social Behavior via Hippuric Acid Metabolism
Source: Neurol Int. 2025 Nov 11;17(11):185. doi: 10.3390/neurolint17110185 (PMC12655642; doi:10.3390/neurolint17110185)
Supplement: Supplementary file 1 [file neurolint-17-00185-s001.zip › neurolint-3930385-supplementary.pdf]

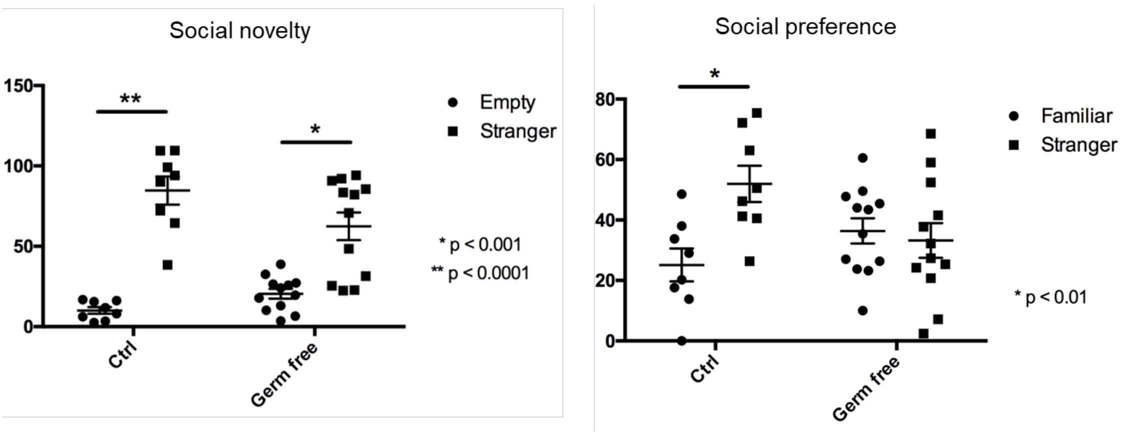

Supplementary Figure S1. Social behavior of germ free mice

Results from social novelty and social preference tests of germ free mice. n=8 (control), 12 (germ free).
